# A live-online mindfulness-based intervention for children living with epilepsy and their families: protocol for a randomized controlled trial of Making Mindfulness Matter©

**DOI:** 10.1186/s13063-020-04792-3

**Published:** 2020-11-11

**Authors:** Klajdi Puka, Karen Bax, Andrea Andrade, Margo Devries-Rizzo, Hema Gangam, Simon Levin, Maryam N. Nouri, Asuri N. Prasad, Mary Secco, Guangyong Zou, Kathy N. Speechley

**Affiliations:** 1 Department of Epidemiology & Biostatistics, Western University, Kresge Building, Room K201, 1151 Richmond Street, London, ON N6A 5C1 Canada; 2 Children’s Health Research Institute, Lawson Health Research Institute, London, Ontario Canada; 3 The Mary J. Wright Research and Education Centre, Western University, London, Ontario Canada; 4 Paediatrics, Western University, London, Ontario Canada; 5 Children’s Hospital at London Health Sciences Centre, London, Ontario Canada; 6 Health Sciences, Western University, London, Ontario Canada; 7 Epilepsy Southwestern Ontario, London, Ontario Canada; 8 Robarts Research Institute, London, Ontario Canada

**Keywords:** Behavioral intervention, Pediatric, Feasibility, Quality of life, Mental health

## Abstract

**Background:**

Epilepsy extends far beyond seizures; up to 80% of children with epilepsy (CWE) may have comorbid cognitive or mental health problems, and up to 50% of parents of CWE are at risk for major depression. Past research has also shown that family environment has a greater influence on children’s and parents’ health-related quality of life (HRQOL) and mental health than epilepsy-related factors. There is a pressing need for low-cost, innovative interventions to improve HRQOL and mental health for CWE and their parents. The aim of this randomized controlled trial (RCT) is to evaluate whether an interactive online mindfulness-based intervention program, Making Mindfulness Matter (M3), can be feasibly implemented and whether it positively affects CWE’s and parents’ HRQOL and mental health (specifically, stress, behavioral, depressive, and anxiety symptoms).

**Methods:**

This parallel RCT was planned to recruit 100 child-parent dyads to be randomized 1:1 to the 8-week intervention or waitlist control and followed over 20 weeks. The intervention, M3, will be delivered online and separately to parents and children (ages 4–10 years) in groups of 4–8 by non-clinician staff of a local community epilepsy agency. The intervention incorporates mindful awareness, social-emotional learning skills, and positive psychology. It is modeled after the validated school-based MindUP program and adapted for provision online and to include a parent component.

**Discussion:**

This RCT will determine whether this online mindfulness-based intervention is feasible and effective for CWE and their parents. The proposed intervention may be an ideal vector to significantly improve HRQOL and mental health for CWE and their parents given its low cost and implementation by community epilepsy agencies.

**Trial registration:**

ClinicalTrials.gov NCT04020484. Registered on July 16, 2019.

## Administrative information

Note: the numbers in curly brackets in this protocol refer to SPIRIT checklist item numbers. The order of the items has been modified to group similar items (see http://www.equator-network.org/reporting-guidelines/spirit-2013-statement-defining-standard-protocol-items-for-clinical-trials/).

**Table:** 

Title {1}	A live-online mindfulness-based intervention for children living with epilepsy and their families: protocol for a randomized controlled trial of Making Mindfulness Matter©
Trial registration {2a and 2b}	ClinicalTrials.gov: NCT04020484. Registered on July 16, 2019. https://clinicaltrials.gov/ct2/show/NCT04020484 All items from WHO Trial Registration Data set can be found within the manuscript.
Protocol version {3}	Version 3.0
Funding {4}	Canadian Institutes of Health Research, Project Grant (PJT-159504)
Author details {5a}	^1^ Epidemiology & Biostatistics, Western University, London, Ontario, Canada ^2^ Children’s Health Research Institute, Lawson Health Research Institute, London, Ontario, Canada ^3^ The Mary J. Wright Research and Education Centre, Western University, London, Ontario, Canada ^4^ Paediatrics, Western University, London, Ontario, Canada ^5^ Children’s Hospital at London Health Sciences Centre, London, Ontario, Canada ^6^ Health Sciences, Western University, London, Ontario Canada ^7^ Epilepsy Southwestern Ontario, London, Ontario, Canada ^8^ Robarts Research Institute, London, Ontario, Canada
Name and contact information for the trial sponsor {5b}	Lawson Health Research Institute 750 Base Line Rd. E, London, ON N6C 2R5 Phone: 519–646-6005 info@lawsonresearch.com
Role of sponsor {5c}	The sponsor played no part in study design; and will play no part in the collection, management, analysis, and interpretation of data; writing of the report; and the decision to submit the report for publication.

## Introduction

### Background and rationale {6a}

Epilepsy is a common debilitating condition characterized by spontaneous, unprovoked seizures. While the prognosis for seizure control is favorable, with 66 to 80% of children becoming seizure-free in the long term [1, 2], it has long been recognized that the impact of epilepsy extends far beyond seizures [3–5]. Unequivocal evidence has shown that up to 80% of children with epilepsy (CWE) may face cognitive, psychiatric, and/or behavioral comorbidities, many of which go under-recognized and untreated, leaving patients with significant unmet mental health needs [6–9]. Importantly, such comorbidities often have a greater negative impact on health-related quality of life (HRQOL) and life outcomes than do epilepsy-specific factors [10–13]. Neuropsychological comorbidities have also been shown to increase the frequency of outpatient visits, emergency department visits, and hospitalizations [14], and negatively impact seizure control after epilepsy surgery, and the response and tolerance to antiseizure medications (ASMs) [15].

It is widely acknowledged that the current standard of care is not adequately addressing emotional and behavioral comorbidities in CWE [6–9]. “Again and again it is pointed out that the comorbidities continue to be under-recognized and undertreated and that patients with epilepsy have significant unmet mental health needs. Nonetheless, there is no indication that this disappointing state of affairs will change anytime soon” [16]. In addition, outpatient pediatric neurology visits are focused on the diagnosis, classification, and treatment of the seizures, without adequate time or other resources to attend to the child’s or parents’ possible unmet needs. This is an important component because families of CWE fare worse than other families on quality of parent-child relationship, parenting confidence, family functioning and stress, and parental psychopathology [3]. Parents of CWE have poorer HRQOL, with up to 50% of mothers of CWE at risk for clinical depression and 58% of parents at risk for anxiety [17–19]. Notably, our findings, along with those of others, point to family environment as an important determinant for long-term HRQOL; epilepsy-related factors, with the exception of seizure control, are seldom associated with HRQOL and mental health [12, 20–22]. It is therefore essential that interventions target parents’ well-being and HRQOL as well as the HRQOL and psychological well-being of CWE.

#### Mindfulness-based interventions

Cognitive-behavioral therapies are increasingly popular in adult health research but their applicability and accessibility limit widespread use in CWE. Cognitive-behavioral therapy and counseling are often administered one-on-one, which is costly and often depend on difficult-to-access specialized therapists; wait times in Ontario, Canada, exceed 6 months [23]. Although there is a risk that the mental health of children and youth can deteriorate while waiting for service, little is done to monitor wait time trends and their impact [23]. Targeting psychiatric and behavioral comorbidities may therefore be best accomplished through community organizations and in group settings. Mindfulness-based interventions may provide an ideal vector to target unmet mental healthcare needs in persons with epilepsy and their families. These group-based interventions are delivered at low cost and by non-medical staff.

Mindfulness-based interventions are effective and have been well-validated for several adult outcomes including physical and mental health, social and emotional well-being, and cognition. Meta-analyses covering a wide spectrum of clinical populations and non-clinical populations report an overall medium effect size of 0.50 to 0.59 across these outcomes [24]. In a recent Cochrane review of psychological interventions for people with epilepsy [25], three studies specifically examined mindfulness-based techniques for adults and determined positive outcomes on mental health, HRQOL, and seizure outcomes [26–28]. There has been far less research on mindfulness with children and youth; the studies that have been done are plagued with methodological limitations including small numbers and lack of randomization or control groups [29]. However, evidence to date indicates that mindfulness interventions for children and youth are feasible, accepted, and enjoyed by participants [30, 31]. The few well-conducted studies on mindfulness interventions in children without physical health issues have reported reduced symptoms of anxiety, depression, and stress, reduced maladaptive coping and rumination, and improved behavioral and emotional self-regulation and focus [29, 30, 32]. Furthermore, a recent systematic review and meta-analysis [29] found that mindfulness interventions were three times more effective (relative to other interventions such as cognitive-behavioral therapy) in alleviating psychological symptoms among children with clinically diagnosed neuropsychological disorders (such as anxiety, learning disability, and externalizing disorders).

In addition to the benefits of programs that deliver mindfulness interventions directly to children, programs that target parents appear to be effective in improving parental functioning and, in turn, promote positive child outcomes [30]. Furthermore, mindfulness-based interventions for parents of children with chronic issues (attention deficit hyperactivity disorder, developmental delays, autism) have been shown to be effective for lessening parental stress and mental health problems. Improvements in parent-child relationships and improved youth behavior management have also been found [33].

Neither the Cochrane review on the impact of psychological treatments for people with epilepsy [25], nor a recent systematic review on mindfulness interventions in youth [29] found studies investigating mindfulness management techniques for CWE. Despite the paucity of studies of mindfulness interventions in childhood epilepsy, there is converging evidence to suggest studying a mindfulness-based intervention in children and families with epilepsy is warranted. There is research pointing to the effectiveness of mindfulness-based interventions on psychological symptoms in adults and children, especially in those with relevant clinical issues similar to CWE [24, 25, 29].

#### Proposed intervention: Making Mindfulness Matter (M3)©

Based on the extant knowledge outlined above, together with a critical review of the benefits of psychological interventions, we propose a low-cost, live online intervention targeting *both* CWE and their parents. The intervention, Making Mindfulness Matter (M3)©, is provided to parents and children and incorporates mindful awareness, social-emotional learning skills, neuroscience, and positive psychology. M3 is delivered by non-clinician staff from a local community epilepsy agency, and integrates attitudes, skills, and behaviors related to mindfulness and social-emotional learning. Mindful awareness is paying attention to our thoughts, feelings, bodily sensations, and surrounding environment in the present moment. Social-emotional learning refers to abilities such as managing thoughts, regulating feelings and behavior, making healthy choices, and taking the perspective of others. The program is delivered over an 8-week period with one 1.5-h session per week for parents and one 1-h session per week for children. Parents and children learn the same core principles: how our brains work, stress and the brain, mindful breathing, mindful sensing, mindful movement, perspective taking, optimism, and gratitude/acts of kindness. Within the parent group, the emphasis is on applying the principles and skills directly to parenting. The child program is designed for children 4–10 years of age, with each lesson including a variety of concrete ways to teach children skills based on their age/developmental level. The program has a standardized protocol, presentation slides, and scripts to be used by the facilitators leading each session. Approximately 80–85% of the program is related to learning about mindfulness, practicing mindfulness, or applying mindfulness. Table 1 provides a description of the topics covered during each M3 session.

**Table 1:** Topics covered during each M3 session

Session	Outline
1	**Topic**: An introduction to the brain, breathing, and mindfulness
	**Description**: The focus of session one is building a comfortable environment and introducing main concepts such as how the brain and our thoughts and feelings work together, mindful awareness, and deep breathing. Parents also learn about neuroplasticity and the STOP model of mindful parenting.
2	**Topic**: How our brains work under stress
	**Description**: Session two teaches how the brain works under stress. Children and parents learn to further identify which part of their brain is busy when they feel big emotions and how mindfulness and a brain break can calm their amygdala, so they can choose to respond, rather than react to stressful situations.
3	**Topic**: Mindful awareness and mindful breathing
	**Description**: The concept of mindfulness is further explored in session 3, with children learning what is mindful or unmindful thinking and practicing how to be in the present moment. Parents learn about the effects of breathing on the brain and body and learn mindful techniques to use with their child.
4	**Topic**: Mindful sensing
	**Description**: Further practice at being in the moment, through mindful sensing, is the focus of session 4. Both parents and children participate in a variety of activities using the five senses mindfully.
5	**Topic**: Mindful movement
	**Description**: Mindful movement is the topic of session 5. Parents learn about the brain-body connection, and mindful awareness of their body and their children’s body during parent-child interactions. Child also learns mindful awareness of their body including how good posture relates to good thinking.
6	**Topic**: Perspective taking
	**Description**: Both parents and children learn how perspective taking is a skill they can practice and strengthen through mindful awareness. Parents explore their child’s perspective through imagining their child is video recording all interactions and using that to understand how they should act in similar situations. Children learn perspective taking through games, books, and video.
7	**Topic**: Choosing optimism and appreciating happy experiences
	**Description**: Choosing optimism and appreciating happy experiences are the focal points of session seven, with parents discovering that optimism can be learned and three techniques to be a more optimistic parent. Children learn about positive and negative thinking, how it affects how we feel and mindful ways to think more positive and have a growth mindset.
8	**Topic**: Expressing gratitude and acts of kindness
	**Description**: Using mindful awareness to practice gratitude and kindness are explored, with children participating in activities that encourage being thankful, and doing acts of kindness for those around them. Parents similarly learn how gratitude and kindness are linked to better mental health and stronger family relationships and that kindness starts with being kind to ourselves.

M3 was modeled after the school-based MindUP program and augmented for provision interactively online and to integrate a parent component. MindUP has been used by over 6 million children in over 12 countries and has been validated in several studies that produced evidence of significant improvement in perspective taking, emotional control, optimism, cognitive control, and stress physiology [34–36]. Children who received the MindUP program also demonstrated greater improvements in self-reported symptoms of depression and peer-rated aggression [34–36]. The low-cost, online group delivery and facilitation by non-clinician staff also allow the program to be scalable to communities across Canada and increases its likely sustainability.

### Objectives {7}

Our long-term goal is to improve the quality of life of children with epilepsy and their family. The objective of this pilot trial is to evaluate the feasibility of utilizing Making Mindfulness Matter (M3)© as a non-medical therapeutic, interactive online intervention for children with epilepsy and their parents. Our central hypothesis is that M3 will be successfully implemented and will improve HRQOL and overall mental health and well-being in CWE and their parents. We plan to test our central hypothesis and accomplish the overall goal of this trial by pursuing the following specific objectives:

Primary objective:

1. To assess the feasibility of successfully implementing Making Mindfulness Matter (M3)© as a live online family intervention for children with epilepsy (CWE) and their parents. Specifically, we will evaluate
  - Study procedures (e.g., recruitment, attrition, time to complete questionnaires),
  - Participant feedback and adherence to M3,
  - Intervention fidelity.

This information will be essential in preparing for a subsequent multi-centered trial across Canada. Feasibility outcomes will be evaluated throughout the course of the study, and the specific outcomes collected and evaluated are presented in section “Outcomes {12}”.

Secondary objectives are to obtain preliminary data regarding the impact of M3 on:

2. Children’s health-related quality of life (HRQOL);
3. Parents’ HRQOL;
4. Children’s (*i*) externalizing problems, (*ii*) internalizing problems, (*iii*) adaptive skills, (*iv*) executive function, and (*v*) severity of epilepsy; and
5. Parents’ (*i*) depression, (*ii*) anxiety, and (*iii*) stress.

All secondary outcomes (i.e., efficacy outcomes) will be evaluated 0–2 weeks before the intervention (i.e., baseline), and 1-week after the intervention (i.e., immediate follow-up). Efficacy outcomes for the intervention arm will also be evaluated 10 weeks after the intervention (i.e., Extended Follow-up). The timeline of assessment is presented in Table 2 and section “Participant timeline {13}”.

**Table 2:** Schedule of evaluations

	Pre-randomization		Post-randomization				
	Screening/consent	Baseline (week: *−1 to 0)*	Intervention *(weeks 1–8)*			Immediate follow-up *(week 9; 1 week post-M3)*	Extended follow-up *(week 18; 10 weeks post-M3)*
			Session 1	Sessions 2–7	Session 8		
Screening form	X						
Informed consent-parent	X						
Assent child			X^a^				
Clinician’s questionnaire		X					
Parent questionnaire							
QOLCE-55		X				X	X
PCGRC						X	X
BASC-3		X				X	X
BRIEF		X				X	X
SF-12		X				X	X
CESD-R		X				X	X
GAD-7		X				X	X
PSI-4-SF		X				X	X
Family APGAR		X				X	X
GASE		X				X	X
Demographics/clinical		X				X	X
Weekly Parent Questionnaire			X	X	X		
Parent Pre-Post Assessment			X		X		
Child Pre-Post Assessment			X^a^		X^a^		
M3 Adherence checklist			X	X	X		
Quality of Implementation			X	X	X		
QOLCE-16 (if participant withdraws)						X	X

*Abbreviations: BASC-3* Behavior Assessment System for Children Scale, 3rd edition, *BRIEF* Behavior Rating Inventory of Executive Function, *CESD-R* Center for Epidemiological Studies Depression, Revised scale, *Family APGAR* Family Adaptability, Partnership, Growth, Affection, and Resolve, *GAD-7* 7-Item Generalized Anxiety Disorder, *GASE* Global Assessment of the Severity of Epilepsy, *PCGRC* Patient-Centered Global Ratings of Change, *PSI-4-SF* Parenting Stress Index, 4th edition Short Form, *QOLCE* Quality of Life in Childhood Epilepsy Questionnaire, *SF-12v2* 12-item Short-Form Health Survey

^a^Will be completed a few days before and a few days after the first and last M3 session

### Trial design {8}

This is a pilot, parallel, partially nested randomized controlled trial (RCT) comparing two arms: intervention (M3) and waitlist control (i.e., treatment as usual). Participants will be randomized 1:1 into the intervention or control arm. This study will not interfere with patients’ clinical care. The intervention will be delivered online to groups of 4–8 on a rolling basis to minimize wait times and allow for timely access to the intervention for the waitlist controls.

## Methods

### Methods: participants, interventions, and outcomes

#### Study setting {9}

Participants will be recruited from the population of children treated for epilepsy in Southwestern Ontario, Canada by the Division of Neurology, Children’s Hospital at London Health Sciences Center (academic hospital) and the solo pediatric neurology practice in Windsor, ON (community clinic). The intervention will occur online.

#### Eligibility criteria {10}

The inclusion and exclusion criteria are listed below. The clinical neurology teams will evaluate inclusion criteria 1–3 and exclusion criteria 1–3; the research coordinator will confirm all eligibility criteria in communication with parents.

##### Inclusion criteria

1. Children aged 4 to 10 years;
2. Diagnosed with epilepsy a minimum of 6 months ago, as per the International League Against Epilepsy operational definition*;
3. Child has reasonable comprehension of spoken language and can follow simple instructions;
4. Child and their parent** are willing to attend all intervention sessions;
5. Child and their parent** have an adequate understanding of English.
  *Operational definition of epilepsy [37]:
6. At least two unprovoked (or reflex) seizures occurring > 24 h apart, or
7. One unprovoked (or reflex) seizure and a probability of further seizures similar to the general recurrence risk (at least 60%) after two unprovoked seizures, occurring over the next 10 years, or
8. Diagnosis of an epilepsy syndrome

  **Parent: refers to parent or guardian self-identifying as most responsible for child’s day-to-day care.

Note: To participate in the online intervention, CWE and their parents would require access to a reliable internet connection and a computer and/or mobile device. All possible efforts will be made to provide families with a mobile device if needed. The proportion of families that require a computer/mobile device and internet connect will be recorded and reported as part of our feasibility outcomes.

##### Exclusion criteria

1. Progressive or degenerative neurological disorder;
2. Other major comorbid non-neurological disorders (e.g., cystic fibrosis, Crohn’s disease, diabetes, renal failure);
3. Scheduled to undergo epilepsy surgery during study period;
4. Child or parent regularly practices complementary health interventions such as meditation;
5. Concurrent enrollment in other intervention trials.

##### Rationale for eligibility criteria

The inclusion criteria, particularly with respect to seizure control, initial levels of HRQOL and mental health (e.g., anxiety, depression, stress), and cognitive ability, are deliberately broad, and we acknowledge that this may limit the possible improvements observed following intervention. However, we believe this is justified given that the primary aim of this trial is to evaluate feasibility and to provide initial data on the potential effect of M3 on key outcomes. The impact of initial levels of HRQOL and mental health problems will be evaluated through a subgroup analyses, which will present an opportunity for a future RCT to be designed to focus on participants with poor HRQOL and mental health. Furthermore, the intervention was modeled after the MindUp program that is designed for all children (not just those with neuropsychological comorbidities).

The age range of 4–10 years was chosen because the intervention has been validated for this age group. We recognize that study results will not generalize to individuals diagnosed with epilepsy in adolescence. Children with newly diagnosed epilepsy (e.g., diagnosed less than 6 months ago) were excluded because this period is marked with increased stress, uncertainty, and changed family dynamic. Children with progressive or degenerative conditions are expected to experience deterioration in health and thus likely in HRQOL over time. Major comorbid non-neurological conditions will most likely continue to have a major impact on HRQOL. Lastly, participants who regularly practice complementary health interventions such as meditation were also excluded. The benefit of introducing M3 to this group was thought to be minimal since such participants are regular practitioners of mindfulness or other complementary health interventions.

#### Who will take informed consent {26a}

Consent is taken by the research coordinator, experienced in working with children and their parents. Parents of eligible children will be provided with a letter of information (LOI) during their clinic visit or via mail, explaining the objectives of the study and all study procedures. A few days later, the research coordinator will contact parents by telephone to discuss the study, answer any questions, and obtain informed consent of those interested. Given the online delivery of the intervention, completing the consent or assent forms in person is not feasible. Parents will provide consent electronically; they will be emailed an individualized link to the consent form through Research Electronic Data Capture (REDCap) [38]. Assent will be obtained from all children aged 5 years and older by a member of the research team over a phone or video call before the first intervention session. The research team member will sign the assent form electronically through REDCap. The research coordinator/assistants are not part of the child’s circle of care.

#### Additional consent provisions for collection and use of participant data and biological specimens {26b}

None / not applicable; there are no plans for ancillary studies not covered by the consent form.

### Interventions

#### Intervention description {11a}

##### Intervention arm: Making Mindfulness Matter (M3)©

Child-parent dyads will participate in a standardized 8-week program, Making Mindfulness Matter (M3)©, described above in section “Proposed intervention: Making Mindfulness Matter (M3)©”. The program will be delivered online using live, interactive sessions to groups of 4 to 8, for 1.5 h each week for the parent group and 1 h each week for the child group. For each group, we will aim to schedule the sessions in consecutive weeks and at the same time and day of the week, though some flexibility in scheduling will be allowed. Children and parents will attend separate, online sessions and at the end of the child session, the parent will be asked to join their child online for a shared mindful exercise. The M3 program will have separate sessions for younger and older children; the content of the sessions will be the same though the activities will be geared towards the participants’ age group. The research coordinator will determine, in discussion with the child’s parents, whether the child is better suited to the younger or older age group depending on the child’s developmental stage and cognitive ability. Typically, the younger group will be composed of children aged 4.00 to 6.99 years and the older group will be composed of children aged 6.00 to 10.99 years.

##### Control arm: waitlist control

Child-parent dyads randomized to the control arm will continue treatment as usual. They will complete the baseline and immediate follow-up questionnaire at comparable times to families in the intervention arm; they will not complete the Extended Follow-up questionnaire. These dyads will be provided with the intervention at the next scheduled session after they have completed the immediate follow-up; the goal is to provide the intervention to controls as soon as possible to avoid differential attrition between the intervention and control arm. During the intervention sessions, participants in the control arm will complete all feasibility questionnaires before and after each session, pertaining to the intervention and their satisfaction with each session.

##### Explanation for the choice of comparators {6b}

Attention placebo control (APC) is preferable over treatment as usual or waitlist control for psychological interventions, to account for non-specific effects a group setting may offer such as social support/social attention [39]. However this design is rarely used in such trials [40]. Two key challenges may explain why the theoretical benefits of APCs have not been realized in practice and informed our decision to not use an APC in this current trial: (1) Disproportional drop-out has been documented; a study assessing a parent training program reported 18% and 50% attrition in the intervention and APC arms, respectively [40]. Furthermore, the reasons for drop-out were different; parents in the APC dropped out without reason or indicating a preference to be in the intervention rather than the APC arm, while parents in the intervention arm dropped out due to scheduling issues. (2) Potential for an ethical issue in a APC arm where participants’ comments and questions in discussions show a risky misunderstanding of key topics related to parenting, which should be addressed but where doing so is outside the protocol for the undirected discussion condition of the APC [40]. We view both these challenges as very likely to occur in the epilepsy population. Given that the aim of the current pilot trial is to evaluate feasibility, we will utilize a waitlist control arm and provide controls with the intervention as soon as possible, to avoid differential attrition between the intervention and control arm. For the subsequent full RCT, we are exploring possible programs to utilize as APC and are considering a cluster random allocation process where all the eligible participants recruited from each institution would be randomized as a group (cluster) to either the intervention or an APC arm where it is much easier to ensure blinding of participants to reduce the likelihood of uneven drop-out. Additionally, a specific a priori protocol could be developed for the APC to incorporate strategies for addressing misinformation about epilepsy.

#### Setting and delivery of the intervention

The program is delivered by non-clinician staff. We have partnered with our local community epilepsy agency, Epilepsy Southwestern Ontario (ESWO), whose staff will be provided the training required to deliver the intervention. Facilitators from ESWO will be trained on the M3 program by the team that created it, led by Dr. Karen Bax. The training will be a 2-day online, interactive session following the standardized M3 training manual. Following the training, the ESWO facilitators will co-lead the program with one established trainer. At the half point of each program, a half-day booster-training session will occur online so that the program facilitators can extend their knowledge of the program and any facilitation issues that arise can be addressed.

The intervention will be delivered online, and a total of four facilitators will deliver each M3 session (two facilitators for the parent group, and two for the child group). Two research assistants will also be present for each online session (one for the parent group, and one for the child group). The role of the research assistants will be to report on intervention fidelity (quality of implementation; described below, in section “Outcomes {12}”) and to assist with the research aspects of the sessions (e.g., facilitating the process of parents completing questionnaires online).

#### Criteria for discontinuing or modifying allocated interventions {11b}

Adverse events, if any, are expected to be minor. There are no planned intervention modifications and no planned circumstances whereby participants will be removed from the intervention by the trial investigators. Participants may withdraw from the study and intervention at any time.

#### Strategies to improve adherence to interventions {11c}

Attendance will be tracked each week; if a session is missed, the research coordinator will contact the parent inquiring about the circumstances and identify if the trial investigators can provide any assistance to avoid missing future sessions. Parents are encouraged to utilize M3 skills outside of the scheduled intervention sessions, by being provided with parent cards that have extension activities (based on the theme of that week) to practice. Adherence to practicing or using the skills outside of the scheduled intervention sessions will be tracked at the start of each session; parents complete a questionnaire inquiring about the utilization of M3 skills or what may have prevented the utilization of those skills (see the supplementary file).

#### Relevant concomitant care permitted or prohibited during the trial {11d}

Usual care for participants continues throughout the trial. There is nothing prohibited.

#### Provisions for post-trial care {30}

Participants randomized to the control arm will be provided with the intervention in the next scheduled session, after they have completed the study. Adverse events, if any, are expected to be minor. Standard care is provided through the Ontario Health Insurance Plan.

#### Outcomes {12}

Each outcome is described below. Feasibility outcomes (primary objective) will be evaluated throughout the course of the study. Efficacy outcomes (secondary objectives) will be evaluated 0–2 weeks before the intervention (Baseline) and 1 week after the intervention (Immediate Follow-up). Secondary outcomes for the intervention arm will also be evaluated 10 weeks after the end of the intervention (Extended Follow-up). The timeline of assessment is presented in Table 2 and section “Participant timeline {13}”.

##### Primary outcome: feasibility

The primary objective of the trial, to evaluate the feasibility of implementing the intervention, will be assessed by evaluating (a) study procedures, (b) participant feedback and adherence to M3, and (c) intervention fidelity, as described below.

Study procedures will be evaluated by tracking (a) the number of patients contacted, (b) participating rate, (c) reasons and/or barriers for non-participation (such as the proportion of families without an internet connection and/or a computer/mobile device), (d) attrition, (e) participant burden (e.g., time to complete questionnaires), and (f) quality of questionnaire data (e.g., the proportion of missing data).

Participant feedback and adherence to M3 will be evaluated using three measures developed for this study (see the supplementary file). The first measure, the Weekly Parent Questionnaire, is completed online by parents at the start and end of each session. At the start of the session it asks about the utilization of M3 skills outside of the scheduled intervention sessions over the past week and whether any adverse events were experienced by the parent or child. At the end of each session, it asks about skills taught in that session and suggestions for improvement. The second measure, Parent Pre-Post Assessment, is completed online by parents at the start and end of the M3 program and evaluates understanding and utilization of the skills taught by M3, as well as participants’ satisfaction with the online program. The third measure, Child Pre-Post Assessment, is completed by children with the help of a research assistant through a video call prior to the first group session and after the program is completed. The measure evaluates children’s understanding of topics discussed in the group, such as how our brain works when we are upset and what mindfulness is. These questions will be read to the children by the researcher and are rated on a 3-point scale as indicated by a smiling, neutral and frowning face.

Intervention fidelity will be evaluated through two measures developed for this study (see the supplementary file). The first measure, M3 Adherence Checklist, is completed online, at the end of each session by facilitators and evaluates whether each planned task/activity was completed, any modifications that were made, problems that arose, and the facilitator's satisfaction with the session and the online format. The measure was designed to evaluate and document how each intervention session ran and was received by participants from the facilitators’ perspective. The second measure, Quality of Implementation, is completed online, at the end of each session by the research assistants present at each session and evaluates the facilitators’ presentation of materials, their confidence, and effectiveness at presenting materials.

##### Children’s health-related quality of life

The parent-reported 55-item Quality of Life in Childhood Epilepsy Questionnaire (QOLCE-55) [41–43] will be used to evaluate the HRQOL of CWE. The QOLCE-55 generates a total HRQOL score and four subscale scores evaluating cognitive, emotional, social, and physical functioning. Scores range from 0 to 100, with higher scores indicative of better HRQOL. The primary interest will be the mean total HRQOL score at the Immediate Follow-up (week 9), adjusting for the total HRQOL score at Baseline (week 0).

Although the QOLCE is a widely used HRQOL scale, no study has calculated the minimum clinically important difference (MCID). Using the methods described by Wiebe et al. [44], we will ascertain meaningful change in HRQOL for the QOLCE-55 using the parent-reported Patient-Centered Global Ratings of Change (PCGRC). This information will allow us to calculate the MCID for the QOLCE-55, and thereby identify the proportion of patients who experience a clinically meaningful change following intervention. The PCGRC consists of 5 items and asks participants to indicate the amount of change experienced relative to baseline in five areas (questions) that correspond to the QOLCE subscales: overall HRQOL, cognitive function (e.g., memory and thinking), emotional well-being, social activities/well-being, and physical activities/well-being. Ratings range from − 7 (a very great deal worse) through 0 (no change) to + 7 (a very great deal better).

If participants withdraw from the study, they will be presented with the option of completing the parent-reported QOLCE-16 [45, 46], an abbreviated version of the QOLCE-55, at the regularly scheduled times (Immediate and/or Extended Follow-up). The QOLCE-16 is composed of 16 items and is a shortened version of the QOLCE-55 and is estimated to take less than 5 min to complete. This will be important to the intention-to-treat analysis in an attempt to follow all randomized participants.

##### Parent’s health-related quality of life

The parent-reported 12-item Short-Form Health Survey (SF-12v2) [47] will be used. It is the most frequently used patient-reported outcome in clinical trials [48] and has been used most frequently in studies evaluating HRQOL of parents of CWE [19]. The SF-12v2 generates a physical and mental health summary score, and 8 subscales: physical functioning, role-physical, bodily pain, general health, vitality, social functioning, role emotional, mental health. The primary interest will be the mean mental and physical health summary score at the Immediate Follow-up (week 9), adjusting for the mean score at Baseline (week 0).

##### Children’s externalizing problems

Externalizing problems will be evaluated using the Externalizing Problems scale of the Behavior Assessment System for Children-Parent Rating Scale (BASC-3-PRS) [49]. This composite scale is composed of the following subscales: hyperactivelity, aggression, and conduct problems. We will utilize the BASC-3 preschool version (validated for children aged 2–5 years) and the BASC-3 child version (validated for children aged 6–11 years). Children with a *T*-score above 59 in a given scale are categorized as “at risk” for that domain. To ensure that parents complete the same version at each follow-up, and given that the study will last approximately 5 months (e.g., 20 weeks), children aged 5.792 years or older will be given the BASC-3 child version, and those aged less than 5.792 years will be given the preschool version at each follow-up. The primary interest will be the mean Externalizing Problems *T*-score at the Immediate Follow-up (week 9), adjusting for the mean score at Baseline (week 0).

##### Children’s internalizing problems

Internalizing problems will be evaluated using the BASC-3 Parent Rating Scale [49] Internalizing Problems scale. This composite scale is composed of the following subscales: anxiety, depression, and somatozation. Children with a *T*-score above 59 in a given scale are categorized as “at risk” for that domain. As explained above, parents of children aged 5.792 years or older will be given the BASC-3 child version, and those parents with children aged less than 5.792 years will be given the preschool version at each follow-up. The primary interest will be the mean Internalizing Problems *T*-score at the Immediate Follow-up (week 9), adjusting for the mean score at Baseline (week 0).

##### Children’s adaptive skills

Adaptive skills will be evaluated using the BASC-3 Parenting Rating Scale [49] composite Adaptive Skills scale. This composite scale is composed of the following subscales: adaptability, social skills, leadership, functional communication, and activities of daily living. Children with a *T*-score above 59 in a given scale are categorized as “at risk” for that domain. As explained above, parents of children aged 5.792 years or older will be given the BASC-3 child version, and parents of children aged less than 5.792 years will be given the preschool version at each follow-up. The primary interest will be the mean Adaptive Skills *T*-score at the Immediate Follow-up (week 9), adjusting for the mean score at Baseline (week 0).

##### Children’s executive function

The parent-reported Behavior Rating Inventory of Executive Function (BRIEF) scale will be used to evaluate children’s self-regulation of cognition, emotion, and behavior [50]. We will utilize the BRIEF-2 version (validated for children aged 5–18 years) and the BRIEF-P (preschool) version (validated for children aged 2–6 years). These measures generate a global executive composite, cognitive regulation index, emotion regulation index, and behavior regulation index. Children with *T*-scores above 60 in a given scale are categorized as “at risk” for that domain. To ensure that parents complete the same version at each follow-up, children aged 5.00 years or older will be given the BRIEF-2, and those aged less than 5.00 years will be given the BRIEF-P version at each follow-up. The primary interest will be the mean global executive composite *T*-score at the Immediate Follow-up (week 9), adjusting for the mean score at Baseline (week 0).

##### Severity of children’s epilepsy

Parents will rate the severity of their child’s epilepsy using the Global Assessment of the Severity of Epilepsy (GASE), a single-item scale measured on a 7-point Likert-type scale [51, 52]. Seven additional items will be asked to validate parents’ response on the GASE; these questions will ask about the severity of seizures, intensity of seizures, falls or injuries during seizures, severity of period immediately after a seizure, amount of antiepileptic drugs, side effects of antiepileptic drugs, and interference of epilepsy or drugs with daily activities. At baseline only, the child’s neurologist will also complete the GASE and the additional seven items. Seizure frequency will also be rated by parents through two questions asking about the number of seizures and number of seizure-free days in the past 30 days. The primary interest will be the parent-reported mean GASE score at the Immediate Follow-up (week 9), adjusting for the mean score at Baseline (week 0).

##### Parent’s depression

The Center for Epidemiological Studies Depression, Revised Scale (CESD-R) is a widely used self-reported checklist evaluating level of depressive symptoms in adults [53, 54]. This 20-item measure generates an overall score for depressive symptoms, ranging from 0 to 60, with scores above 16 indicating risk for depression. The primary interest will be the mean depression score at the Immediate Follow-up (week 9), adjusting for the mean score at Baseline (week 0).

##### Parents’ anxiety

The 7-item Generalized Anxiety Disorder (GAD-7) scale evaluates parents’ self-reported symptoms of anxiety [55]. The GAD-7 generates an overall anxiety score ranging from 0 to 21, with scores above 10 indicating moderate-severe anxiety. The primary interest will be the mean anxiety score at the Immediate Follow-up (week 9), adjusting for the mean score at Baseline (week 0).

##### Parents’ stress

The Parenting Stress Index 4 Short-Form (PSI-4-SF) – screens for stress in the parent-child relationship, identifying dysfunctional parenting and child adjustment problems [56]. This 36-item parent-reported scale generates scores on three domains (Parental Distress, Parent-Child Dysfunctional Interaction, and Difficult Child), which combine to form a Total Stress scale. Scores in the 81st percentile or higher are indicative of high stress. The primary interest will be the mean Total Stress score at the Immediate Follow-up (Week 9), adjusting for the mean score at Baseline (week 0).

##### Sociodemographic and clinical characteristics

Parents will be asked to complete several questions on key demographic characteristics of the family (such as parents’ age, education, occupation, marital status) and children (such as gender, age, and comorbidities), similar to questions we have used successfully in previous studies [12]. Parents will also complete the Family Adaptability, Partnership, Growth, Affection, and Resolve (Family APGAR) [57, 58]. This scale evaluates satisfaction with family relationships in the following areas: coping with problems, partnership, growth, affection, and resolve; scores range from 0 to 10.

At baseline, neurologists will complete a short questionnaire documenting clinical characteristics of each patient’s epilepsy: severity of epilepsy, seizure type and frequency, type of epilepsy syndrome, age at onset and diagnosis, medication information, adverse effects, and other comorbid conditions. Type of seizure and epilepsy or epileptic syndrome will be classified using the International League Against Epilepsy’s 2017 classifications [59, 60].

##### Adverse events

At the start of each M3 session parents are asked whether they experienced any physical discomfort, emotional discomfort or distress, or an increase in problems in relationships with others, while practicing any of the M3 skills in the past week (see supplementary file). Parents are also asked if their child experienced any unwanted negative events while practicing any M3 skills in the past week.

##### Rationale for lack of child-reported measures

The majority of questionnaires will be completed by parents, with few exceptions (e.g., clinicians’ report on clinical characteristics, and children’s pre-post assessment of M3 skills). We acknowledge that children’s own perceptions are important and that this trial will not capture children’s perceptions of the intervention and its impact; however, we believe this is reasonable for three reasons. First and foremost, the primary objective of this pilot RCT is not to evaluate effectiveness as reported by children; the primary aim is to evaluate feasibility, focusing on participant adherence and facilitators’ adherence and quality of implementation. Second, questionnaires are completed at home and given the children’s age (4–10 years), parents would undoubtably have to provide the child with the questionnaires and likely assist, potentially biasing their responses. Lastly, given the children’s age (4–10 years), the choice of validated self-reported questionnaires is limited and it has been proposed that children under the age of 8 years may lack the cognitive maturity and verbal comprehension capacity to provide self-reports [61]. We are evaluating ways to best incorporate children’s self-reports in a subsequent large-scale RCT of M3.

##### Rationale for short follow-up period

A short follow-up period was chosen for two reasons. First, the primary objective of this pilot RCT is not to evaluate the long-term effectiveness of M3. Second, the short follow-up serves to minimize attrition in the control arm in two ways: by providing controls with the intervention as soon as possible, thereby keeping them interested and engaged in the study, and second it allowed for blinding of participants and facilitators to the study hypotheses, as described below, in section “Who will be blinded {17a}*”*. The intention is to evaluate longer term outcomes in a subsequent large-scale RCT.

#### Participant timeline {13}

Parents will be able to complete all questionnaires online via REDCap, or if preferred, using mailed questionnaires. The baseline and follow-up questionnaires are estimated to take 90 min to complete. Parents will also be asked to complete a brief online questionnaire via REDCap at the start and end of each M3 session pertaining to the content of the M3 sessions and their experience with the intervention.

**Fig. 1:**
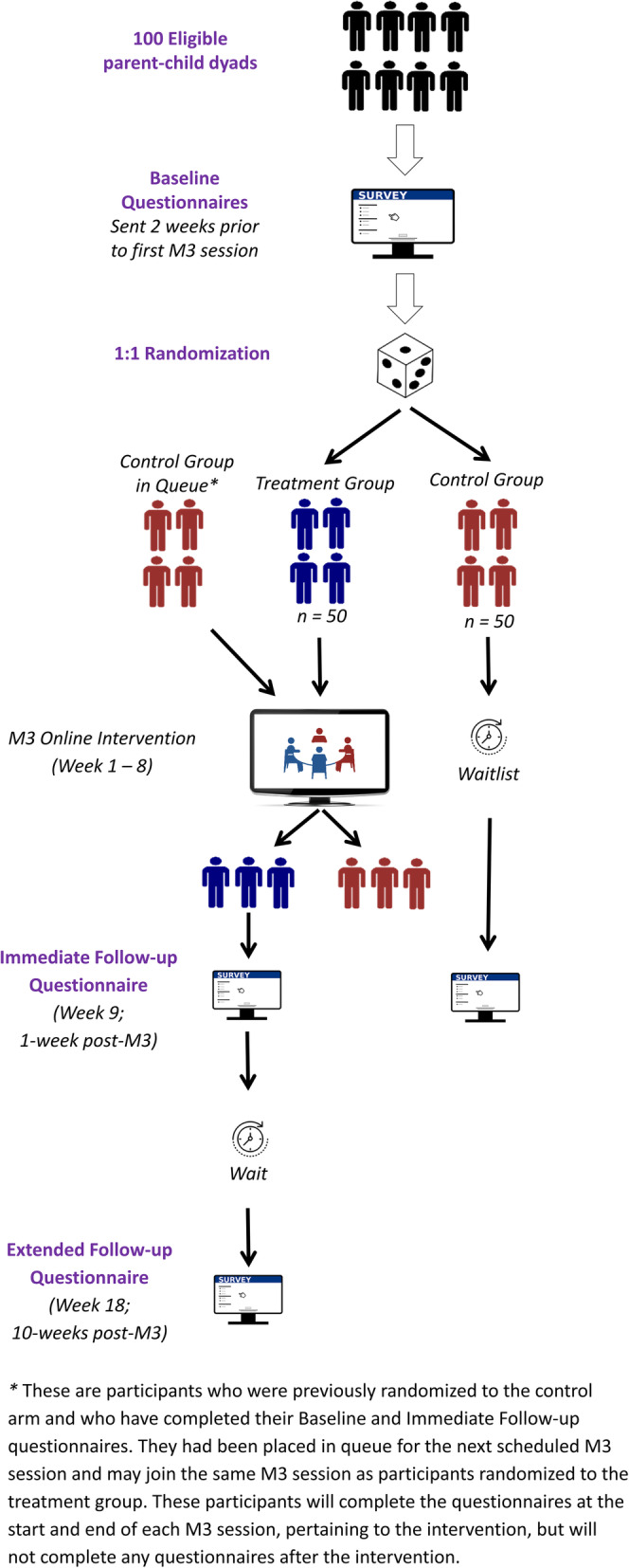
Schematic of participant progress

Figure 1 highlights participants’ progression through the study. Two weeks prior to the first M3 session of the intervention arm, participants will be sent the baseline questionnaire and asked to complete it within 1 week, which will allow for a 1-week buffer window prior to the first M3 session for the intervention arm. Once the Baseline questionnaire is completed, participants will be randomized. One week after the last M3 session for the intervention arm, all participants will be sent the Immediate Follow-up questionnaire. Participants will be asked to complete the questionnaire within 1 week, with the goal of receiving all completed questionnaires within 2 weeks.

Participants in the intervention arm will be asked to complete a similar questionnaire, the Extended Follow-up, 10 weeks after the end of the M3 session. For participants in the control arm, the Immediate Follow-up questionnaire is the final follow-up questionnaire they complete. To provide interested controls with the intervention as soon as possible, those interested in receiving the intervention will be placed in a queue and will be able to join the next scheduled M3 sessions. Therefore, M3 sessions may be composed of participants randomized to the intervention arm and/or controls who have completed the study (completed Baseline and Immediate Follow-up questionnaires). During the M3 sessions, all participants will complete the same online questionnaires pertaining to the content of the M3 sessions and their experience with the intervention. Once the intervention ends, those who were randomized to the control arm will not need to complete any follow-up questionnaires. Therefore, the role of the controls in the intervention would be to simply honor the commitment for everyone to receive the M3 sessions and to “fill” the group (given that 4–8 participants are needed per group). The advantage of including dyads randomized to intervention and dyads previously randomized to the control arm is that it will increase the pool of participants waiting to receive the intervention and thereby expedite the time it takes to fill each group. The other advantage is that it would ensure that dyads randomized to the control group will receive the intervention as soon as possible and ameliorate attrition in the control group.

#### Sample size {14}

The sample size calculation was based on the results of our pan-Canadian prospective cohort study of children (aged 4–12 years) with newly diagnosed epilepsy [45]. Two years after diagnosis, we observed the mean score on the QOLCE-55 to be 76.4 with a standard deviation of 14.5. To our knowledge, there are no estimates of minimal clinically important difference (MCID) for the QOLCE-55, and no intra-class coefficient estimates available for the impact of group-based psychological interventions in CWE. We therefore assumed a conservative ICC among participants in M3 of 0.10, an average cluster size of 6, and aimed to detect a large effect size (*d* = 0.8) [62], corresponding to a 11.6-point difference on the QOLCE-55 between the two study arms. The 11.6 point difference is in line with the MCID found for the adult equivalent HRQOL measure, the QOLIE-31 [44]. Using the method for a trial with a group-administered intervention, a control arm with independent subjects, and 1:1 randomization [63], the study requires 38 child-parent dyads in the intervention arm and 38 child-parent dyads in the control arm to have 80% power and the 2-sided 5% significance level. We will aim to recruit 100 child-parent dyads to account for a liberal estimate of 24% attrition.

#### Recruitment {15}

Participants will be recruited from the population of children treated for epilepsy in Southwestern Ontario by the Division of Pediatric Neurology, Children’s Hospital at London Health Sciences Center and the single pediatric neurology practice in Windsor, ON. The clinical neurology teams will identify all patients meeting clinical eligibility criteria and will provide their parents with a letter of information (LOI) that explains the aims of the study and all study procedures. The LOIs will be distributed by mail or occasionally in person during the child’s regularly scheduled epilepsy appointment for those patients due for an appointment in the near future. A member of the research team will subsequently contact parents in a few days to explain the study objectives and procedures, answer any questions, confirm eligibility, and obtain informed consent.

### Assignment of interventions: allocation

#### Sequence generation {16a}

Stratified block randomization will be used. Allocation tables were developed by KP in STATA 13.1 through the *ralloc* command using an allocation ratio of 1:1, randomly permuted blocks of varying sizes, and stratified by children’s age group (younger vs. older). We stratified by child's age group because the intervention will have separate sessions for younger and older children, as described in section "Intervention arm: Making Mindfulness Matter (M3)©".

#### Concealment mechanism {16b}

Randomization will be facilitated centrally through REDCap. The allocation sequence was generated and uploaded to REDCap by KP, who is not involved with participant recruitment, scheduling or any other forms of participant contact. This web-based system prevents circumvention of the randomization process and ensures allocation concealment.

#### Implementation {16c}

Randomization is stratified based on the child’s age group, as described in section “Sequence generation {16a}”. Randomization will occur as close as possible to the intervention to minimize the delay between study arm allocation and the delivery of the intervention. Therefore, once informed consent is received, the parent-child dyad will enter the queue for randomization. Given that the intervention will be delivered in groups of 4 to 8, and assuming some attrition and scheduling conflicts, once 12 to 16 participants per stratum are in queue for randomization and available for the scheduled date/time of the intervention, participants may begin the study by receiving the Baseline questionnaires (as described in the paragraph below). If there are controls who have completed the study and are in the queue to receive the intervention, at least 9 participants need to be in queue for randomization. This would ensure that a minimum of 4 participants are randomized to the control arm, which is important to fulfill our obligations to participants by providing all participants with the opportunity to receive the intervention. It would be ethically problematic if, for example, one participant is randomized and they are allocated to the control arm; this participant cannot be offered the intervention at the end of the study because at least 4 participants are needed to run the intervention sessions.

Once enough participants are ready to be randomized, those in the queue for randomization will receive an individualized link to the baseline questionnaire and electronic gift card via email 2 weeks before the first scheduled M3 session of the intervention arm. These participants will have 2 weeks to complete the questionnaire but will be asked to complete it within a week, to allow for a 1 week buffer. A member of the research team will also connect with the parent and child to attain assent of the child, complete the child Pre Assessment, and offer a short tutorial of how to use Zoom (the video conferencing application) if the parent is unfamiliar with this platform. Upon completion of the online baseline questionnaire by parents, the research coordinator will randomize participants via REDCap. Those randomized to the intervention (and controls who are in the queue for the intervention) will be provided with instructions on how access the online sessions. Those randomized to the control arm will be told that they will be placed in the next scheduled M3 sessions, after they complete the Immediate Follow-up questionnaire. Participants who were unable to complete the baseline questionnaire will be placed in the next scheduled group of participants to be randomized. After the M3 program is completed, the Research Coordinator will contact the family again to have the child complete the Post Assessment.

### Assignment of interventions: blinding

#### Who will be blinded {17a}

Blinding of participants and program facilitators (i.e., the staff delivering the M3 intervention) will be difficult given the nature of the intervention. However, blinding to study hypotheses will be achieved by explaining to participants and program facilitators that the study aims to evaluate the effectiveness of the intervention given to all participants; however, some participants will receive the intervention in the upcoming M3 session, whereas others will receive the next scheduled M3 sessions. In so doing, we hope to eliminate any biases associated with the participants’ and program facilitators’ perception or biases of being assigned to the intervention or control arm. The data analyst evaluating efficacy outcomes (e.g., HRQOL, mental health) will be blinded.

#### Procedure for unblinding if needed {17b}

There is no requirement for emergency unblinding procedures.

### Data collection and management

#### Plans for assessment and collection of outcomes {18a}

All data and questionnaires are captured online using REDCap. If needed, participants may request mailed questionnaires, which would be subsequently entered into REDCap by a member of the research team. Feasibility outcomes are collected throughout the course of the study (e.g., recruitment, randomization, retention, and the assessment procedures) by the research team. After each intervention session, the research assistant and facilitators will complete questionnaires on REDCap designed to assess adherence and quality of implementation. Facilitators will be emailed individualized links to the questionnaire through REDCap, whereas research assistants will be able to enter data directly on REDCap. On the day of an intervention session, participants will be emailed an individualized link through REDCap with the questionnaire they must complete before or at the start of the session and at the end of the session; these questions pertain to the content of the M3 sessions and their experience with the intervention.

Similarly, with respect to efficacy outcomes (Baseline, Immediate Follow-up, and Extended Follow-up questionnaire), participants will be emailed (through REDCap) an individualized link to the questionnaire along with an individualized link to a $25 gift card.

Individualized links will also be emailed through REDCap to clinicians who would complete a short questionnaire pertaining to the patient’s clinical characteristics.

#### Plans to promote participant retention and complete follow-up {18b}

If a session is missed, the research coordinator will contact the parent inquiring about the circumstances and identify if the trial investigators can provide any assistance to avoid missing future sessions. If randomized participants are not able to attend the M3 sessions, but would still be interested in completing the Immediate and/or Extended Follow-up questionnaires, they would be encouraged to do so. If participants withdraw, the reasons will be recorded and they will be presented with the option of completing the parent-reported QOLCE-16 [45, 46], an abbreviated version of the QOLCE-55, at the Immediate and/or Extended Follow-up, that is estimated to take less than 5 min to complete. This will be important to the intention-to-treat analysis in an attempt to follow all participants randomized into one of the two study arms.

#### Data management {19}

All data are entered onto REDCap [38], a widely utilized and secure web-based platform. The research team evaluating the quality of implementation and other study data undergo a 1.5-h training session and REDCap training. Data are held in a secure server at Lawson Health Research Institute, London, Ontario, Canada. REDCap is also utilized to randomize and email individualized questionnaire links to participants. Participants will not be required to provide an answer to each question. A number of quality control procedures in REDCap were specifically created for this project and will be utilized, such as providing participants with an ignorable warning that some questions have been left blank to minimize the frequency of missing questions. Other quality control procedures include providing warning or reminder messages to the research team (e.g., indicating that a participant is not eligible), preventing certain actions (e.g., preventing the randomization of participants unless all inclusion criteria are met and the Baseline questionnaire is completed), notifying the research team if adverse events are reported or if questions have been left blank, and tracking participants’ progression through the study and the time it took participants to complete each questionnaire. Once participants have completed their questionnaire, a research assistant will be notified by REDCap and subsequently review the responses and follow-up with participants if needed. If the participant requested paper questionnaires, the data will be reviewed and entered in REDCap by a research assistant.

#### Confidentiality {27}

REDCap servers follow hospital security guidelines and policies and all the web-based information transmission is encrypted. REDCap was developed specifically around Health Insurance Portability and Accountability Act (HIPAA)-Security guidelines and is recommended to researchers by Privacy Offices and Institutional Review Boards of many organizations.

#### Plans for collection, laboratory evaluation, and storage of biological specimens for genetic or molecular analysis in this trial/future use {33}

No biological samples will be collected.

### Statistical methods

#### Statistical methods for primary and secondary outcomes {20a}

Descriptive statistics will be run with continuous variables summarized using means and standard deviations and categorical variables summarized using frequencies and proportions. To evaluate study feasibility, descriptive statistics will be used to summarize study procedures among all participants, including (a) number of families contacted, (b) participation rate, (c) reasons and/or barriers for non-participation, (d) attrition rate, (e) time to complete questionnaires, and (f) missing data on questionnaires. To evaluate (1) participant feedback and adherence to M3 and (2) intervention fidelity, data from the intervention and control arms will be combined and descriptive statistics used to describe responses to the Weekly Parent Questionnaire, Parent Pre-Post Assessment, and Child Pre-Post Assessment, M3 Adherence Checklist, and Quality of Implementation questionnaire.

To evaluate Efficacy Outcomes (e.g., HRQOL), we will use linear mixed models, which are suitable for group-administered interventions to account for the clustering in the intervention arm and no clustering in the control arm [63, 64]. The primary interest will be comparing the mean scores of the intervention and control arms at the Immediate Follow-up (e.g., HRQOL 1-week post-M3), adjusting for potential imbalance at Baseline (e.g., HRQOL pre-M3). Given that age group (younger vs. older) was a variable used in stratifying randomization, analyses will adjust for age group.

Similarly, generalized linear mixed models will be used to evaluate whether the intervention and control arms differ with respect to the proportion who show clinically significant improvement, and the proportion with clinically elevated symptoms post-intervention [65].

#### Interim analyses {21b}

There will be no interim analyses.

#### Methods for additional analyses (e.g., subgroup analyses) {20b}

Two sensitivity analyses are planned. First, to evaluate whether outcomes at the Immediate Follow-up (1 week post-M3) are retained or changed over the longer term (10-weeks post-M3), linear mixed models will be used to evaluate outcomes at each time point (Baseline, Immediate Follow-up, Extended Follow-up) for participants randomized to the intervention arm. Second, we do not plan to adjust for any covariates (other than age group and the value of the outcome variable at baseline), but may consider adjusting for variables that are substantively imbalanced between the study arms within the sensitivity analyses.

Lastly, adverse events will also be evaluated. Data from the intervention and control arms will be combined and descriptive statistics will be used to evaluate the frequency of adverse events, based on parents’ reports at the start of each M3 session. All collected adverse events will be reported.

#### Methods in analysis to handle protocol non-adherence and any statistical methods to handle missing data {20c}

Analysis will follow the intention-to-treat principle. We do not plan to impute missing values, but may consider use of multiple imputation or other strategies within the sensitivity analysis.

#### Plans to give access to the full protocol, participant-level data, and statistical code {31c}

The current document outlines the full protocol. Non-identifiable data may be made available in response to a reasonable and well-motivated request to the principal investigator. Data cannot, however, be made freely available to the public, due to privacy regulations and informed consent. A data sharing agreement will also need to be signed.

### Oversight and monitoring

#### Composition of the coordinating center and trial steering committee {5d}

The research team is multi-disciplinary, spanning epidemiology (KNS, KP), biostatistics (GZ, KP), pediatric neurology (AA, CC, MDR, HG, SL, MNN, ANP), clinical psychology (KB), and knowledge translation (MS). The immediate trial team (KS, KB, KP, and research coordinators) will oversee all aspects of the study, such as internal monitoring of the trial data and training of staff. The immediate trial team meets weekly or more frequently as needed to oversee the project and monitor progress. KB, a clinical psychologist, will be automatically made aware (via REDCap notifications) of any adverse events reported by parents and, if needed, will contact and follow-up with participants.

#### Composition of the data monitoring committee, its role and reporting structure {21a}

The immediate trial team (KS, KB, KP, and research coordinators) meets weekly or as needed to oversee the project and monitor progress. The main goal will be to evaluate study procedures and identify the need for any adjustments (such as recruitment procedures). Given that adverse events, if any, are expected to be minimal, an independent data monitoring committee was not deemed necessary.

#### Adverse event reporting and harms {22}

As indicated previously, adverse events, if any, are expected to be minor. At the start of each M3 session parents will be asked whether they experienced any physical discomfort, emotional discomfort or distress, or an increase in problems in relationships with others, while practicing any of the M3 skills in the past week. Parents will also be asked if their child experienced any unwanted negative events while practicing any M3 skills in the past week. KB, a clinical psychologist, will be automatically made aware of any adverse events reported by parents and, if needed, will contact and follow-up with participants. With respect to reporting adverse events, data from the intervention and control arms will be combined and descriptive statistics will be used to evaluate the frequency and severity of adverse events. All collected adverse events will be reported.

#### Frequency and plans for auditing trial conduct {23}

No audits have been planned. The immediate trial team (KS, KB, KP, and research coordinators) meets weekly or as needed to oversee the project and monitor progress.

#### Plans for communicating important protocol amendments to relevant parties (e.g., trial participants, ethical committees) {25}

Version control using protocol identifies and dates will be used. The research ethics board will approve amendments and substantive changes will be noted on clinicaltrials.gov and/or subsequent journal publication(s).

##### Past protocol versions

This manuscript is based on protocol version 3.0. In Version 3.0, the intervention was modified to be delivered interactively online, as a result of the COVID19 pandemic. Twelve participants were randomized prior to protocol version 3.0 and participated in the in-person intervention. Data from these participants will not be combined with future participants who complete the intervention online. A new randomization list was generated for participants participating in the online intervention, and a total of 100 will be randomized.

In Version 2.0, the operational process was modified for a pragmatic reason to increase the pool of participants waiting to receive the intervention and thereby expedite the time it takes to fill each group (as described in section “Participant timeline {13}”). The original process (in Version 1.0) was more restrictive and used a stringent timeline, requiring that the intervention arm receives the intervention in weeks 1–8 and the control arm receives the intervention in weeks 10–17. This limited the participant pool because eligible participants had to be available during weeks 1–8 and weeks 10–17 (given that they could have been randomized to either group).

#### Dissemination plans {31a}

We will develop a publication and dissemination plan to include conference presentations and journal publications. We plan to write to all participants to inform them of the trial results. We will also plan dissemination to relevant patient and clinical interest groups.

## Discussion

This protocol describes a funded pilot RCT evaluating the feasibility of implementing an interactive online mindfulness-based intervention for CWE and their parents. To the best of our knowledge, this is the first behavioral intervention targeting young children with epilepsy which is particularly important as evidence to date suggests that early identification and treatment of epilepsy comorbidities are essential as there may be a window of opportunity for early intervention in some children [66, 13]. Interventions must be implemented early before problems become entrenched and interfere with the development of basic cognitive, behavioral, and social skills crucial for long-term educational, vocational, and interpersonal adaptation. In addition, interventions (such as M3) targeting self-regulation and executive functioning may be most effective during the preschool and early school years given the ongoing brain development and maturation of children in this age group [67, 68]. M3 is also unique because the intervention is delivered to parents and children, providing parents and their child with a common vocabulary and skillset, and enabling them to jointly practice the skills learned. Lastly, we believe that this program provides an ideal vector to target the unmet mental health care needs of CWE and their parents, given that the program may be delivered by non-clinical staff and is delivered online in a group setting. These characteristics would allow the intervention to be implemented at low cost and by epilepsy support centers, allowing scalability to communities across the country and increasing its likely sustainability.

The primary goal of this RCT is to evaluate feasibility, with respect to participant adherence and the quality with which facilitators are able to implement the intervention. Additionally, the feedback received from the recruitment, randomization, retention, and assessment procedures, as well as the feedback from parents and facilitators will allow us to better implement this intervention in a subsequent, larger multicenter trial. The data collected will also allow for the calculation of the MCID in HRQOL scores and enable better estimates of the impact of the intervention on key outcomes. This information will be necessary for robust sample size calculations. In reaching these feasibility goals, this study will employ a short follow-up period, comparing the intervention and control arm immediately after the intervention. This short follow-up may make it difficult to identify significant improvements in HRQOL, which may take some time to develop. However, participants in the intervention arm will also report on outcomes 10 weeks after the intervention, to estimate a longer-term effect. Improvements in other domains, such as depression, anxiety, executive function, and stress, may be evident in the short term. Regardless, the information obtained from these measures will be essential in the development of our subsequently large-scale RCT.

### Trial status

Recruitment is ongoing. Potential participants were first identified and entered on REDCap on November 12, 2019, and LOI were first mailed to participants on November 28, 2019. The protocol was modified for online delivery of the intervention on September 21, 2020, and the first online intervention session is anticipated on October 2020. The trial is scheduled to end on September 2022.

## Supplementary information

**Additional file 1.** Study Specific Questionnaires and forms

## Data Availability

Not applicable. This manuscript does not contain any data.

## Abbreviations

ASM: Antiseizure medication
BASC-3: Behavior Assessment System for Children Scale, 3rd edition
BRIEF: Behavior Rating Inventory of Executive Function
CESD-R: Center for Epidemiological Studies Depression, Revised Scale
CWE: Children with epilepsy
Family APGAR: Family Adaptability, Partnership, Growth, Affection, and Resolve
GAD-7: 7-Item Generalized Anxiety Disorder
GASE: Global Assessment of the Severity of Epilepsy scale
HRQOL: Health-related quality of life
LOI: Letter of information
M3: Making Mindfulness Matter
MCID: Minimal clinically important difference
PCGRC: Patient-Centered Global Ratings of Change
PSI-4-SF: Parenting Stress Index, 4th edition Short Form
QOLCE: Quality of Life in Childhood Epilepsy Questionnaire
RCT: Randomized controlled trial
REDCap: Research Electronic Data Capture
SF-12v2: 12-Item Short-Form Health Survey
