# Supplementary material for: A live-online mindfulness-based intervention for children living with epilepsy and their families: protocol for a randomized controlled trial of Making Mindfulness Matter©
Source: Trials. 2020 Nov 11;21:922. doi: 10.1186/s13063-020-04792-3 (PMC7657360; doi:10.1186/s13063-020-04792-3)
Supplement: Supplementary file 1 — Additional file 1. Study Specific Questionnaires and forms [file 13063_2020_4792_MOESM1_ESM.pdf]

Western
Mary J. Wright Research and Education Centre at Merrymount

Children's HEALTH RESEARCH INSTITUTE

Western

Schulich MEDICINE & DENTISTRY

Group#__________ Session/Week:_________ Participant ID#________________________________

Parent Weekly Questionnaire

<u>Please complete the following questions before the start of this group:</u>

1. Did you or your child practice a M3 skill since our last session?

   ☐ Yes → (go to a)
   ☐ No → (go to 2)

   a) If yes, about how many times did you or your child practice a M3 skill?

You:	Your Child:	You and Your Child Together
☐ 1-3	☐ 1-3	☐ 1-3
☐ 4-6	☐ 4-6	☐ 4-6
☐ 7-10	☐ 7-10	☐ 7-10
☐ 10+	☐ 10+	☐ 10+

   b) How many times did you and your child practice a breathing break this week?

   ____

   c) What situation led up to using the M3 skill?
   ☐ To practice ☐ Due to frustration ☐ Due to anger
   ☐ other_____________

   d) Did you/your child find it helpful?
   You: Yes ☐ No ☐ Your Child: Yes ☐ No ☐

2. If no, what kept you from practicing M3 skills this week?

____

____

3) While practicing any of the M3 skills this past week, did you experience any of the following events:

a) Physical discomfort (aches, pains etc.)

- ☐ No
- ☐ Yes, please explain: ____

____

b) Emotional discomfort or distress.

- ☐ No
- ☐ Yes, please explain: ____

____

c) An increase in problems in relationships with others (e.g. child(ren), partner, co-worker).

- ☐ No
- ☐ Yes, please explain: ____

____

4) While practicing any of the M3 skills this past week, did your child experience any unwanted negative events

- ☐ No
- ☐ Yes, please explain: ____

____

____

5) In the past 7 days, how many seizures did your child have? ____________
(# of seizures)

6) In the past 7 days, how many seizure-free days did your child have? ______________
(# seizure-free days)

Group#__________ Session/Week:_________ Participant ID#________________________________

Please complete the following questions AFTER the group:

1. What information/skills discussed today do you think will be helpful for you as a parent?

   Why?

2. Were their certain parts of our session today that you felt we should have spent more time, or less time on?

3. Were there any parts of the session that you found confusing? Why? (E.g., not enough time, difficult concept).

4. Additional comments/feedback?

Parent Pre Assessment

ID Code: ______________________   Date: ___________________________
(DD-MMM-YYYY)

Based on the last 8 weeks, please answer the following statements by circling the response that best fits your opinion. There are no right or wrong answers. For example:

I eat bread	Never	Sometimes	Often (circled)	Regularly

1. I practice mindfulness.	Never	Sometimes	Often	Regularly
2. My child and I practice mindfulness together.	Never	Sometimes	Often	Regularly
3. I think about the brain and how it connects to our emotions and behaviours when supporting my child.	Never	Sometimes	Often	Regularly
4. I am confident in my ability to help my child calm down when she/he is upset.	Never	Sometimes	Often	Regularly
5. I use calm down tools in helping my child calm down when she/he is are upset.	Never	Sometimes	Often	Regularly
6. I am able to manage my own emotions when supporting my child's needs.	Never	Sometimes	Often	Regularly
7. I can recall and reflect on happy experiences between myself and my child	Never	Sometimes	Often	Regularly
8. I am able to be in the moment with my child when parenting with limited distractions.	Never	Sometimes	Often	Regularly
9. I am able to recognize in my body when I am feeling stressed.	Never	Sometimes	Often	Regularly
10. I am able to look at my parenting from the perspective of who my child is and why she/he is doing what they are doing.	Never	Sometimes	Often	Regularly
11. I feel confident in my ability to be firm but kind when supporting my child.	Never	Sometimes	Often	Regularly

Western Mary J. Wright Research and Education Centre at Merrymount | Children's Health Research Institute | Western | Schulich Medicine & Dentistry

Parent Post Assessment

ID Code: ____________________ Date: _________________________
(DD-MMM-YYYY)

Based on the last 8 weeks, please answer the following statements by circling the response that best fits your opinion. There are no right or wrong answers. For example:

I eat bread	Never	Sometimes	Often (circled)	Regularly

1. I practice mindfulness.	Never	Sometimes	Often	Regularly
2. My child and I practice mindfulness together.	Never	Sometimes	Often	Regularly
3. I think about the brain and how it connects to our emotions and behaviours when supporting my child.	Never	Sometimes	Often	Regularly
4. I am confident in my ability to help my child calm down when she/he is upset.	Never	Sometimes	Often	Regularly
5. I use calm down tools in helping my child calm down when she/he is are upset.	Never	Sometimes	Often	Regularly
6. I am able to manage my own emotions when supporting my child's needs.	Never	Sometimes	Often	Regularly
7. I can recall and reflect on happy experiences between myself and my child	Never	Sometimes	Often	Regularly
8. I am able to be in the moment with my child when parenting with limited distractions.	Never	Sometimes	Often	Regularly
9. I am able to recognize in my body when I am feeling stressed.	Never	Sometimes	Often	Regularly
10. I am able to look at my parenting from the perspective of who my child is and why she/he is doing what they are doing.	Never	Sometimes	Often	Regularly
11. I feel confident in my ability to be firm but kind when supporting my child.	Never	Sometimes	Often	Regularly

Based on the last 8 weeks, please answer the following statements by selecting the response that best fits your opinion. There are no right or wrong answers.

	Very Dissatisfied	Dissatisfied	Neutral	Satisfied	Very Satisfied
12. How would you rate your overall satisfaction with the M3 program?	☐	☐	☐	☐	☐

	Strongly Disagree	Disagree	Neither agree nor disagree	Agree	Strongly Agree
13. I found this program informative	☐	☐	☐	☐	☐
14. I found this program to be helpful	☐	☐	☐	☐	☐
15. I found the online program easy to use	☐	☐	☐	☐	☐
16. I was happy to use the computer to access the M3 intervention	☐	☐	☐	☐	☐
17. The online format made it easier to attend each session	☐	☐	☐	☐	☐
18. I was happy with the amount of interaction and engagement in the program	☐	☐	☐	☐	☐
19. I would recommend this on-line program to a friend	☐	☐	☐	☐	☐

20. What did you most like about the online program?

____

____

____

____

21. What did you least like about the online program?

____

____

____

Western Mary J. Wright Research and Education Centre at Merrymount | Children's Health Research Institute | Western | Schulich Medicine & Dentistry

Child Pre and Post Assessment

Date: ________________

ID Code: __________________

Yes I don't know No

1. I know how my brain works when I am angry or upset.

2. I know what a breathing break is.

3. I use breathing breaks to calm my big feelings.

4. I have lots of great ideas to help me when I have a problem.

5. I know how to be kind to others.

6. Mindfulness is…

____

M3 Adherence Checklist. Session # ____

Facilitator Names: ____
Date: (day/month/year)________________________________ Group Number:___________________
Person(s) filling out this form :____________________________ ☐ Parent Group ☐ Child Group
# of Participants:__________________

Did you complete each activity below?						
**Task 1:** ***[Task name goes here]***	**Task 2:** *[Task name goes here]*	**Task 3:** *[Task name goes here]*	**Task 4:** *[Task name goes here]*	**Task 5:** *[Task name goes here]*	**Task 6:** *[Task name goes here]*	**Task 7:** *[Task name goes here]*
☐ Yes Completely<br>☐ Yes with changes<br>☐ No	☐ Yes Completely<br>☐ Yes with changes<br>☐ No	☐ Yes Completely<br>☐ Yes with changes<br>☐ No	☐ Yes Completely<br>☐ Yes with changes<br>☐ No	☐ Yes Completely<br>☐ Yes with changes<br>☐ No	☐ Yes Completely<br>☐ Yes with changes<br>☐ No	☐ Yes Completely<br>☐ Yes with changes<br>☐ No
If you made changes, please describe the here, or if you did not complete an activity, please describe why here.						

________Total # of activities competed out of 7
________Total # of activities not completed

How would you rate your overall satisfaction with:

	Very Dissatisfied	Dissatisfied	Neutral	Satisfied	Very Satisfied
Today's on-line session overall	☐	☐	☐	☐	☐
The interaction and engagement of the participants	☐	☐	☐	☐	☐
The online format of the session	☐	☐	☐	☐	☐
Ease of use of delivering the intervention online	☐	☐	☐	☐	☐

Which activities listed above did the participants seem well engaged in and why?

____

____

____

Which activities did the participants not seem engaged in and why?

____

____

____

M3-E RCT

QUALITY OF IMPLEMENTATION

□ **Parent Group** □ **Child Group**

Group Number: ______________________ Date: ________________________
(dd/mm/yyyy)

Session #: ___________________________ # of Participants:_____________________

Your name (observer): ____

Instructions

The following questions assess the overall quality of the M3 session and delivery of the information. You should complete the form after observing the entire session. Prior to this, you should read through the questions to become familiar with them. You may also find it helpful to take notes during the observations – for example, each time a facilitator provides an explanation you could put a checkmark beside the appropriate rating. Use your best judgment and do not circle more than one response. *NOTE:* this questionnaire will be entered into REDCap; the responses will not seen by the program facilitators.

1. How prepared were the facilitators for the session?

1<br>Not Prepared	2	3<br>Somewhat Prepared	4	5<br>Very Prepared

1- Materials not ready or not available, facilitators did not arrive on-line early, facilitators were not familiar with activities
3 - Some materials ready, facilitators not fully prepared to start session on time, facilitators familiar with some of the activities
5 - Materials ready and set up for activities, facilitators early to on-line session, facilitators very familiar with activities

2. In general, how clear were the program facilitators' explanations of activities?

1<br>Not clear	2	3<br>Somewhat clear	4	5<br>Very clear

1- Most participants did not understand instructions and could not proceed, many questions asked
3- About half of the group understood, while the other half asked questions for clarification
5- 90-100% of the participants began and completed the activity/discussion with no hesitation and no questions

3. To what extent did the facilitators keep track of time during the session and activities?

1<br>Not on time	2	3<br>Some loss of time	4	5<br>Well on Time

1- Facilitator did not have time to complete the material (particularly at the end of the session), regularly allowed discussions to drag on (e.g., participants seem bored or began discussing non-related issues in small groups)
3- Missed a few points, sometimes allowed discussions to drag on
5- Completed all content of the session, completed activities and discussions in a timely manner (using the suggested time limitations in the program manual, if available)

4. To what extent did the presentation of materials seem rushed or hurried?

1<br>Very rushed	2	3<br>Somewhat rushed	4	5<br>Not rushed

1- Facilitators didn't allow time for discussion, didn't have time for examples, told participants that they were in a hurry, body language suggested stress or hurry
3- Some deletion of discussion/activities, sometimes stated but does not explain material
5- Didn't rush participants or speech but still completed all the materials, appeared relaxed

5. To what extent did the participants appear to understand the material?

1<br>Little understanding	2	3<br>Some understanding	4	5<br>Good understanding

Use your best judgment based on participant conversations and feedback.
Roughly: 1 - Less than 25% seemed to understand; 3 - About half; 5 - 75-100% understood

6. How actively did the group members participate in discussions and activities?

1<br>Little Participation	2	3<br>Some participation	4	5<br>Active participation

Use your best judgment based on listening to the discussions and feedback.
Roughly, 1 - Less than 25% participated; 3 - About half participated; 5 - 75-100% participated

7. How well did the facilitators manage participation?

1<br>Poor	2	3<br>Average	4	5<br>Excellent

1 - Did not elicit participation from quiet participants, did not manage dominant talkers, group was loud and unfocused
5 - Able to draw out quiet participant, managed dominant talkers, group remained focused and on task

8. How well did facilitators manage the use of technology to present the session material?

1<br>Poor	2	3<br>Average	4	5<br>Excellent

1 –Screen sharing was not smoothly introduced, the use of break out rooms or the chat function was confusing and not conducive to learning.5 - Screen sharing was smoothly introduced, the use of break out rooms or the chat function was well managed and conducive to learning

9. How well did the facilitators support and encourage participants.

1<br>Poor	2	3<br>Average	4	5<br>Excellent

1 – During activities, facilitators not actively involved with participants, did not support contributions or accomplishments of participants, very limited use of open-ended questions
3 – Limited use of open-ended questions, used subjective or evaluative language (I like it, you're smart)
5 – During activities, facilitators were actively involved with participant, supported contributions or accomplishments with specific and non-evaluative language (I can tell that you put a lot of thought into your project), frequently used open-ended questions

10. Using the scale provided, rate the facilitators on the following qualities:

a) Knowledge of the program

1<br>Poor	2	3<br>Average	4	5<br>Excellent

1 – Could not answer questions, mispronounced names; read from the manual
5 - Provided information above and beyond what's in the manual, seemed very familiar with the concepts, answered questions with ease

b) Ability to communicate goals and "key points" of the session

1<br>Poor	2	3<br>Average	4	5<br>Excellent

1 – Didn't state the goals of the session, didn't summarize key messages for activities, or they were unclear
5 – Clearly stated the goals of the session, provided key take away messages for each activity

c) Level of enthusiasm

1<br>Poor	2	3<br>Average	4	5<br>Excellent

1 - Presented information in a dry and boring way, lacked personal connection to material, appeared "burned out"
5 - Made clear that the program is a great opportunity, got participants talking and excited, was outgoing

d) Poise and confidence

1<br>Poor	2	3<br>Average	4	5<br>Excellent

1 - Appeared nervous or hurried, did not have good eye contact
5 - Did not hesitate in addressing concerns, was well organized, not nervous

e) Rapport and communication with participants

1<br>Poor	2	3<br>Average	4	5<br>Excellent

1 – Didn't remember names, did not "connect" with participants, acted distant or unfriendly
5 - Got participants talking and excited, very friendly, used people's names when appropriate, seemed to understand the community and its needs

f) Effectively addressed questions/concerns

1<br>Poor	2	3<br>Average	4	5<br>Excellent

1 - Engaged in "power struggles," responded negatively to comments, gave inaccurate information, didn't direct participants elsewhere for further information if needed
5 - Answered questions of fact with information and questions of value with validation, if didn't know the answer was honest about it and directed them elsewhere

g) Provided a welcoming on-line atmosphere

**1**<br>**Poor**	**2**	**3**<br>**Average**	**4**	**5**<br>**Excellent**

1 – Did not greet participants, little to no eye contact, stiff instead of animated body language, negative tone of voice, hard to see on the screen (only see part of their face or body)
5 – Greeted all participant, made eye contact with participant when addressing them, smiled, showed open body language, easily seen on the screen, , used warm tone of voice, used respectful language

h) Effectively transitioned from one activity to another

**1**<br>**Poor**	**2**	**3**<br>**Average**	**4**	**5**<br>**Excellent**

1 – Did not use transitions or transitions were unclear and confusing.
5 – Used transitions to connect one activity to another, transitions were smooth and clear.

i) Effectively managed the emotional climate

**1**<br>Poor	2	3<br>Average	4	5<br>Excellent

1 – Emotional climate was negative, negative behaviors were not mediated, comments with bias were not addressed
5 – Emotional climate was positive, respectful and fun; negative behaviors and biased comments were mediated, or there was no evidence of bias but rather a mutual respect and inclusion of others

11. Rate the overall quality of the program session.

**1**<br>**Poor**	**2**	**3**<br>**Average**	**4**	**5**<br>**Excellent**

Summary measure of all the preceding questions - assesses the extent of material covered and performance of the facilitator.

Excellent sessions look like:
- *Participants were doing rather than talking about activities*
- *Non-judgmental responses to questions*
- *Answering questions of fact with information, questions of value with validation*
- *Good time management and well organized*
- *Adequate pacing—not too fast and did not drag*
- *Used effective checks for understanding.*
- *Supportive and encouraging towards participants.*

Poor sessions look like:
- *Lecture-style presenting of content*
- *Reading the content from the notebook*
- *Stumbled along with the content and failing to make connections to what has been discussed previously or what participants are contributing.*
- *Uninvolved participants*
- *Got into power struggles with participants about the content.*
- *Judgmental responses*
- *Flat affect and boring style*
- *Unorganized and random*
- *Lost track of time*

12. Please note at least one major strength of the session and/or facilitators' delivery of the material.

LETTER OF INFORMATION FOR PARENTS/GUARDIANS

Study Title: Making Mindfulness Matter in Children with Epilepsy (M3 Epilepsy)
Principal Investigator: Kathy Nixon Speechley, Ph.D.
[Contact Details]

Funder: Canadian Institutes of Health Research

1. Invitation to Participate

As co-principal investigators, Dr. Kathy Nixon Speechley and Dr. Karen Bax are inviting you and your child who has epilepsy to participate in research about the health and mental health benefits of program that teaches mindful awareness and social-emotional skills because we know that epilepsy can negatively impact quality of life for children and their families.

2. Why is this study being done?

The purpose of this study is to determine whether or not a live online group program called Making Mindfulness Matter can benefit children with epilepsy and their parents. Making Mindfulness Matter (M3) teaches mindful awareness and social-emotional skills in an online group setting to learn to pay better attention to our feelings, thoughts and behaviours in the moment so that we can better manage strong emotions, make positive choices, and feel less stress. There is very little research that describes how a program like this could support well-being for both children and parents.

The study aims to evaluate M3 as a family support and determine whether the M3 program has a positive effect on quality of life and well-being for children with epilepsy and their parents. This letter outlines the procedures for the study. If you agree to participate, you will be asked to sign the consent form.

3. How long will you be in this study?

The M3 program is 8 weeks long, with a 1.5 hour online group session each week for parents and a 1-hour online group session for children. The group sizes are small (4 to 8 people), and children and parents will be in separate sessions and come together in a combined segment at the end of each child session. In addition to the M3 program, you will be asked to complete questionnaires two or three times: at the start of the study andat 8 weeks and, for half of you, 18 weeks later. Therefore, the total duration of the study will be 18 weeks. The questionnaires are estimated to take 90 minutes to complete.

4. What will happen during the study?

If you decide to participate, you will be "randomized" to either begin the program right away or 10 or more weeks later (depending on when the next group session will be scheduled). Randomization means that you are put into a group by chance (like flipping a coin). You will have an about 50:50 chance of starting the program right away or 10 or more weeks later, there is no way to predict when you will begin the program. Neither you nor the researchers can choose if you will begin the program right away or 10 or more weeks later. Your epilepsy care will continue as usual throughout the entire course of the study.

The study is designed for children aged 4 to 10 years who were diagnosed with epilepsy at least 6 months ago including those who are still having seizures as well as those who are seizure-free and their parent/guardian.

5. What are the study procedures?

After consent, you will be asked to complete questionnaires at two time points: at the start of the study and 8 weeks afterwards. Those who begin the program right away, will complete an additional questionnaire18 weeks after the first. Most questions ask about your child and some questions are about you, as the parent/caregiver. There are also demographic questions about your household. Questions will ask about your child's experiences, current quality of life, stress, behavior and feelings. At the start of the study, your child's neurology clinician will complete a one-page questionnaire to provide the researchers with information about your child's epilepsy such as type and severity.

You will also be asked a few questions at each group session to gather additional feedback about the M3 program and your experience with mindfulness. Some examples include, "Did you or your child practice a mindful skill since our last session?" and "Were there any parts of the session you felt were confusing?

Just prior to starting the program and following the last session, your child will be asked to complete a "feeling face" questionnaire about topics discussed in the group such as how our brain works when we are upset and what is mindfulness. The questions will be read to the children by the researcher and the children will respond online with the face that best represents how they feel.

Both you and your child may choose to not answer any question you wish. None of the answers you or your child provide will contain any personal information (e.g., name) that could be used to identify you or your child. , You will complete these online questionnaires at home. You will be provided with an individualized link to the questionnaire through email.

Regardless of when you begin the M3 program, your epilepsy care will continue as usual.

6. What are the possible risks of participating in this study?

The potential risks associated with participation in the present study are low. If you experience stress while participating in the study, you may find it beneficial to access this website describing strategies for handling stress: https://childdevelopmentinfo.com/family-living/stress/#.WJtAdG8rK70. During the M3 sessions, facilitators will be available to discuss strategies related to stress reduction, and you will be asked weekly whether you are experiencing any increase in stress as a result of the program. Referrals to other resources will be provided if needed.

While there is no guarantee, we will do our best to protect the privacy of all your information by using only a study number and not including your name on any questionnaires or forms that might allow you to be individually identified.

7. What are the benefits?

A benefit of this study is that it provides an opportunity to experience the potential benefits that mindful awareness may have for you and your child's health and mental health (e.g., decreased feelings of stress, more focused attention in the moment, better management of strong emotions), although it is possible that you will not experience any direct benefits by participating.

8. Is participation voluntary?

Participation is voluntary including speaking to the researchers and you may also withdraw your participation in the research study and/or your child's at any time without any negative consequences. If you decide to

withdraw your own and/or child's participation from the study, you will have the choice of whether the information that was collected prior to you leaving can still be used in the study.

9. How will information be kept confidential?

Your identity and that of your child will be kept confidential in any reports or presentations that result from the study. The responses from the questionnaires about you and your child will be put into a computer database called REDCap that is password protected and only accessible by study staff. REDCap servers follow hospital security guidelines and policies and all the web-based information transmission is encrypted. REDCap was developed specifically around privacy guidelines and is recommended to researchers by Privacy Offices and Institutional Review Boards of many organizations. Your name, telephone, address, e-mail and full date of birth will also be kept securely in REDCap and on a master list so we can contact you and administer the study. Any written information that identifies you/your child will be kept in a locked filing cabinet in the research office.

According to Lawson Health Research Institute policy, collected information will be kept for 15 years and then the computer file will be permanently deleted and the consent forms in the file will be shredded. Representatives of the University of Western Ontario Research Ethics Board or Lawson Health Research Institute may require access to your study-related records to monitor the conduct of the research.

10. Are participants compensated for being in the study?

Should you choose to participate you will receive a $25.00 gift card each time you are asked to complete the questionnaire at the start of the study and at 8 weeks. Those who are asked to complete a follow-up questionnaire will receive an additional $25.00 gift card at 18 weeks.

11. What are the rights of the participants?

You have the right to not answer individual questions about you and your child. You do not waive any legal rights by providing consent to participate.

12. Whom do participants contact for questions?

If you would like more information about this project, please contact Dr. Speechley by phone XXX-XXX-XXX or by email XXXXXXXXXX. If you have any questions about your rights as a research participant or the conduct of this study, you may contact the Patient Relations Office at LHSC at (519) 685-8500 ext. 52036 or access the online form at: https://apps.lhsc.on.ca/?q=forms/patient-relations-contact-form.

In a few days, a member of our research team will telephone you to answer any questions you may have about the study and find out whether you wish to participate. If you agree, you will be provided a link to consent on-line through REDCap.

Sincerely,

Kathy Nixon Speechley, Ph.D. Karen Bax, Ph.D., C. Psych.

ASSENT LETTER

Study Title: Making Mindfulness Matter in Children with Epilepsy (M3 Epilepsy)

Principal Investigator: Kathy Nixon Speechley, Ph.D.
[contact details]

1. Why are you here?

Epilepsy can make life harder. You are being invited to be part of a study to see if there are ways that may make living with epilepsy easier.

2. Why are we doing this study?

The aim of the study is to see if a mindful awareness group program can help children with epilepsy and their parents feel better. Mindful awareness simply means noticing what is happening right now. The live online group program teaches things like being able to notice what is happening right now, learning to control big emotions and being positive.

3. What will happen to you?

If you want to be in the study, you will join the online child program for 8 weeks with other children. At another time, your Mom or Dad will join the online parent program. At the end of each session your Mom or Dad will come in your group and practice a mindful skill with you.

4. Will there be any tests?

No, there will not be any tests. There will be questions for your parents to answer about how you are doing. At the beginning of the class and 8 weeks later, we will ask you some questions on how you feel about what you learned. At the start of the study, your doctor will answer questions about your health.

5. Do you have to be in the study?

You do not have to be in the study. No one will be mad at you if you do not want to do this. If you do not want to be in the study, tell the program leader or your parents. Even if you say yes, you can change your mind later.

6. What if you have questions?

You can ask questions at any time, now or later. You can ask your family or the program leader or contact Dr. Kathy Speechley.

Assent Form

Study Title: Making Mindfulness Matter in Children with Epilepsy (M3 Epilepsy)

Principal Investigator: Kathy Nixon Speechley, Ph.D.
[Contact Details]

Assent form was discussed and the child agreed to participate. ☐Yes ☐ No

Person obtaining assent ___________________________

Signature of person obtaining assent _______________________________

Date assent was obtained _______________________
